# Functional delivery of lncRNA *TUG1* by endothelial progenitor cells derived extracellular vesicles confers anti-inflammatory macrophage polarization in sepsis via impairing miR-9-5p-targeted SIRT1 inhibition

**DOI:** 10.1038/s41419-021-04117-5

**Published:** 2021-11-06

**Authors:** Wentao Ma, Weihong Zhang, Bing Cui, Jing Gao, Qiuhong Liu, Mengying Yao, Hanbing Ning, Lihua Xing

**Affiliations:** 1 Department of Respiratory Intensive Care Unit, First Affiliated Hospital of Zhengzhou University, Zhengzhou, 450052 P.R. China; 2 Department of Anatomy, School of Nursing and Health College, Zhengzhou University, Zhengzhou, 450001 P.R. China; 3 Department of Nephrology, First Affiliated Hospital, Henan University of Traditional Chinese Medicine, Zhengzhou, 450052 P.R. China; 4 Department of Digestive Diseases, First Affiliated Hospital of Zhengzhou University, Zhengzhou, 450052 P.R. China

**Keywords:** Cell biology, Diseases

## Abstract

The delivery of biomolecules by extracellular vesicles (EVs) derived from endothelial progenitor cells (EPCs) has been proven to ameliorate sepsis, yet the therapeutic mechanism remains to be elucidated. Taurine upregulated gene 1 (*TUG1*) is a long noncoding RNA (lncRNA) that is downregulated in sepsis. The current study was designed to explore the role of EPCs derived EVs transmitting *TUG1* in macrophage polarization and macrophage-mediated inflammation in a cecal ligation and puncture (CLP)-induced sepsis mouse model. *TUG1* was underexpressed in CLP-induced sepsis, and its reexpression induced anti-inflammatory macrophage polarization and suppressed macrophage-medicated inflammatory injury to the pulmonary vascular endothelium. EPCs derived EVs transmitted *TUG1* to promote M2 macrophage polarization. Luciferase, RIP, and RNA pull-down assays showed that *TUG1* could competitively bind to microRNA-9-5p (miR-9-5p) to upregulate the expression of sirtuin 1 (SIRT1). Furthermore, EPCs derived EVs transmitted *TUG1* to promote M2 macrophage polarization through the impairment of miR-9-5p-dependent SIRT1 inhibition. Finally, EPCs derived EVs carrying *TUG1* were verified to ameliorate sepsis-induced organ damage in the murine model. In summary, EPCs derived EVs transmit *TUG1* to attenuate sepsis via macrophage M2 polarization. This study also highlights the proinflammatory mechanism associated with miR-9-5p-mediated inhibition of SIRT1, which contributes to a more comprehensive understanding of the pathogenesis of sepsis.

## Introduction

Sepsis is a dysfunctional systemic inflammatory disorder initiated by microbial infection, which frequently results in organ failure and mortality [1]. The associated high mortality and need for expensive treatment bring urgency to the search for more effective sepsis therapies [2, 3]. Interestingly, profiling of host-derived inflammatory biomarkers may be of great value to distinguish etiologically different types of infections [4]. Besides, macrophage activation presents as a vital immune dysfunction in organs remote to a local injury, which ultimately manifests in systemic complications [5]. Therefore, we contend that molecular investigations focused on immune dysfunction should aid in the development of protective therapies against sepsis.

Recent research has demonstrated the functionality of extracellular vesicles (EVs) as key carriers of novel diagnostic molecules or preventative targets for septic patients, and therefore can be used for the development of individualized therapies [6]. EVs derived from the endothelial progenitor cells (EPCs) intensify the protective effects against sepsis-induced microvascular dysfunction [7]. The term EV encompasses exosomes, microvesicles, and other vesicles that can convey cargos of lipids, peptides, RNAs, and sugars essential for various cellular processes in inflammatory conditions including the immune response, and coagulopathy [7, 8]. Noncoding RNAs (ncRNAs) are a broad category of long noncoding RNA (lncRNAs) and microRNAs (miRs) that can be packaged in EVs and thus serve as specific biomarkers for the degree of acute kidney injury, especially sepsis [9, 10]. On the basis of a whole blood transcriptomic investigation, lncRNA taurine upregulated gene 1 (*TUG1*) has been identified as one of the five lncRNAs most relevant to sepsis [11]. Several researchers have demonstrated the downregulation of *TUG1* in sepsis and its potential as an inhibitor of sepsis-induced inflammation and acute kidney injury [12]. In view of the aforementioned literature, we aimed at substantiating whether EVs derived from EPCs can transmit lncRNA *TUG1* to protect against sepsis-induced inflammatory damage to the pulmonary vascular endothelium. According to the proposed mechanism of competing endogenous RNA (ceRNA), transcripts have miR binding sites that compete for posttranscriptional regulation [13]. A recent study proposed that the ceRNA regulatory network could be used to investigate mechanisms underlying the pathophysiology of sepsis [14]. Interestingly, the ceRNA network of *TUG1* and microRNA-9-5p (miR-9-5p) has been identified to be involved in the progression of human osteosarcoma [15]. As predicted by the Starbase website, miR-9-5p was identified to be a miR bound by *TUG1*, while sirtuin 1 (SIRT1) was regarded as a target of miR-9-5p. SIRT1, which is a protein deacetylase of the NAD^(+)^-dependent class III, is commonly implicated as a key immune mediator through its effects on the expression of various proinflammatory cytokines such as TNF-α, while also affecting the activation of macrophages [16, 17]. Strikingly, downregulation of SIRT1 by miR-9 intensified lipopolysaccharide (LPS)-induced inflammation [18]. Hence, we explored whether miR-9-5p and SIRT1 participate in the protective role of EVs shuttled *TUG1* in sepsis.

## Materials and methods

### Animals and cell culture

Male C57BL/6 mice were purchased from the Hunan SJA Laboratory Animal Co., Ltd (Hunan, China) and acclimatized in a specific-pathogen-free environment at a temperature of 20–22 °C with 40–60% relative humidity and free access to water and food, under a 12 h light/dark cycles for at least 1 week before experimentation. These mice (aged 6–8 weeks, weighing about 20–25 g) were selected to establish the cecal ligation and puncture (CLP)-induced septic mouse model. The experiments involving mice were performed with approval of the Animal Ethics Committee of the First Affiliated Hospital of Zhengzhou University. Adequate measures were taken to minimize the number of included animals in the experiments and their suffering.

The mouse-derived macrophage cell line RAW264.7 was purchased from the Cell Resource Center, Shanghai Institutes for Biological Sciences, Chinese Academy of Sciences (Shanghai, China). The RAW264.7 cells were cultured using Dulbecco’s modified Eagle’s Medium/F12 (Wisent Biotechnology, Nanjing, China) supplemented with 10% fetal bovine serum (Coring, Australia), 100 IU/mL penicillin and 100 μg/mL streptomycin (Sigma-Aldrich, Munich, Germany) under saturated humidity at 37 °C and 5% CO_2_. RAW264.7 cells were exposed to 500 ng/mL LPS. The complementary DNA (cDNA) sequence of *TUG1* was amplified using PfuUltra II Fusion HS DNA polymerase (Stratagene, Agilent Technologies, Santa Clara, CA), and then inserted into the pcDNA3.1 vector (Invitrogen, Carlsbad, CA) to generate the overexpression vector pcDNA-TUG1. The siRNA (si)-negative control (NC), si-SIRT1 (50 nM), mimic control (50 nM), miR-9-5p mimic (50 nM), inhibitor control (150 nM), miR-9-5p inhibitor (150 nM), and miR-204 mimic (50 nM) were provided by Ribobio (Guangzhou, China). EPCs were transfected with pcDNA-NC and pcDNA-TUG1 using Lipofectamine 3000 (Invitrogen, Carlsbad, CA).

Macrophages were transfected with the corresponding mimic NC, miR-9-5p mimic, pcDNA-NC, pcDNA-TUG1, si-NC, si-SIRT1, and miR-204 mimic (50 nM) according to the provided kit instructions.

## Results

### Overexpression of *TUG1* alleviates symptoms in septic mice

**Fig. 1:**
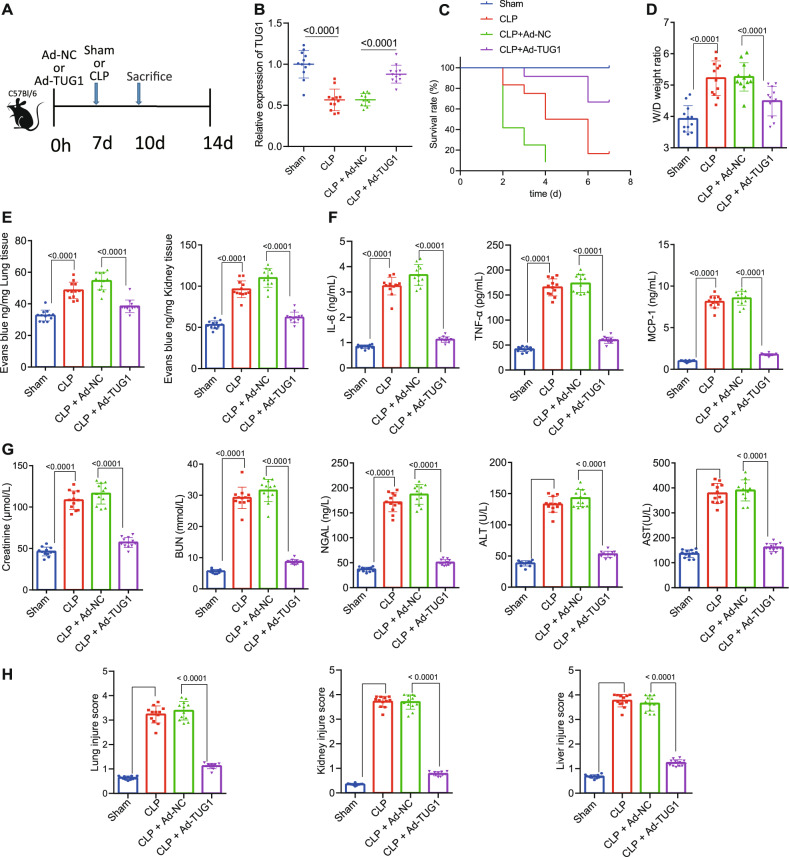
Upregulated *TUG1* suppresses inflammation in the pulmonary vascular endothelial cells and alleviates sepsis in mice. **A** Flow chart of septic mice treated with Ad-*TUG1* vector. **B** The expression of *TUG1* in the lung tissues of septic mice determined by RT-qPCR. **C** The survival rate of mice within 7 days after treatment. **D** Pulmonary edema in septic mice assessed by the wet/dry ratio. **E** The measurement of blood vessel leakage in lung and liver of septic mice using Evans blue dye. **F** Serum levels of IL-6, TNF-α, and MCP-1 were determined by ELISA. **G** Determination of creatinine, BUN, NGAL, AST, and ALT levels in mouse serum. **H** The quantitative analysis of tissue damage scores in lung, liver, and kidney tissues. Measurement data were expressed as mean ± standard deviation. One-way ANOVA was adopted for comparison between multiple groups followed by Tukey’s post hoc test. Kaplan–Meier analyses were performed to calculate the survival rate. Log-rank test was conducted for univariate survival analysis. *p < 0.05 vs. sham-operated mice; ^#^*p* < 0.05 vs. CLP-operated mice treated with Ad-NC (*n* = 12).

As shown in Table 1, several lncRNAs associated with the vascular pathology of sepsis have been identified [19]. Among those lncRNAs, *TUG1* is reported to principally impair miR-34b-5p-mediated downregulation of GAB1, thereby restraining sepsis-triggered acute kidney injury [12]. To further investigate the regulatory role of *TUG1* in sepsis, CLP-induced septic mouse models were established, and the Ad-*TUG1* or Ad-NC vector was injected into the mice via the tail vein 1 week before modeling, with 12 mice per group (Fig. 1A). We found that the expression of *TUG1* was decreased in the septic mice receiving CLP. Besides, mice injected with the Ad-*TUG1* showed increased *TUG1* expression (Fig. 1B). After the 7-day observation, the survival rate of sham-operated mice was 100%, while the survival rate of CLP-operated mice was lower as compared to the sham-operated mice. The survival rate of CLP-operated mice injected with Ad-*TUG1* was higher than those injected with Ad-NC (Fig. 1C). Meanwhile, the pathological changes revealed aggravated pulmonary edema (Fig. 1D), and increased pulmonary and renal vascular leakage in the mice receiving CLP surgery (Fig. 1E), accompanied with elevated levels of creatinine, BUN, NGAL, AST, ALT (Fig. 1G), IL-6, TNF-α, and MCP-1 (Fig. 1F). However, pretreatment with Ad-*TUG1* resulted in the suppression of the aforementioned disturbances and elevated expression of several sepsis indicators in the CLP-operated mice (Fig. 1D–G). We also found that Ad-*TUG1* treatment resulted in a reduction of visible tubule vacuoles in the kidney outer medulla, brush border loss and diminished inflammatory cell infiltration, and suppressed vacuolation in the liver tissues of the CLP-treated mice (Fig. 1H).

**Table 1:** LncRNAs related to sepsis.

LncRNAs	Expression in sepsis	Target gene	Model
MALAT1	Up	miR-125b	Mice
NEAT1	Up	miR-204	Humans in vitro
NEAT1	Up		Humans
NEAT1	Up		Mice
NEAT1	Up	–	Humans
Lnc-ANRIL	Up	–	Humans
Lnc-ANRIL/miR-125a axis	Up		Humans
HOTAIR	Up		Rats in vitro
HOTAIR	–		Rats
LncRNA H19	Down		Humans in vitro
LncRNA ITSN1-2	Up		Humans
HULC	Up	–	Mice in vitro
UCA1			
*TUG1*	Down		Humans in vitro
TapSAKI	Up		Rats in vitro
HOTAIR	Up	–	Mice
MALAT1	Up	–	Mice in vitro
MALAT1 and EZH2	Up	–	Rats in vitro

*Lnc/LncRNA* long noncoding RNA, *miR* microRNA, *MALAT*1 metastasis-associated lung adenocarcinoma transcript 1, *NEAT*1 nuclear paraspeckle assembly transcript 1, *ANRIL* antisense noncoding RNA in the INK4 locus, *HOTAIR* HOX transcript antisense RNA, *ITSN1*-2 RNA intersectin-2, *HULC* highly upregulated in liver cancer, *UCA*1 urothelial carcinoma-associated 1, *TUG1* taurine upregulated gene 1, *TapSAKI* transcript predicting survival in AKI, *EZH*2 enhancer of zeste homolog 2.

### *TUG1* contributes to the polarization of M1 to M2 macrophages

**Fig. 2:**
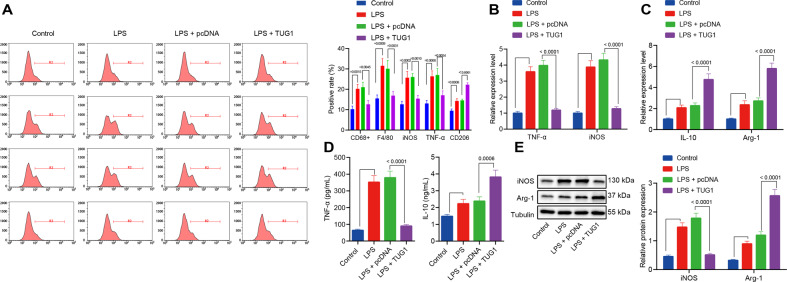
*TUG1* induces macrophage polarization into an anti-inflammatory M2 state. **A** The number of CD68^+^ and F4/80^+^ macrophages assessed by flow cytometry and percentages of M1 markers iNOS and TNF-α as well as M2 marker CD206 in a single-cell suspensions of whole lung tissue from the mice (*n* = 12). **B** The mRNA expression of M1 markers iNOS and TNF-α in macrophages treated with LPS determined by RT-qPCR. **C** RT-qPCR for determination of mRNA expression of M2 markers IL-10 and Arg-1 in macrophages treated with LPS. **D** The contents of IL-10 and TNF-α protein in medium examined by ELISA. **E** The protein expression of M1 markers iNOS and Arg-1 in macrophages treated with LPS measured by western blot analysis. Measurement data were expressed as mean ± standard deviation. One-way ANOVA was adopted for comparison between multiple groups followed by the Tukey’s post hoc test. Cell experiments were conducted three times independently. *p < 0.05 vs. control macrophages; ^#^*p* < 0.05 vs. LPS-induced macrophages treated with pcDNA.

Macrophage polarization is a key process in the pathogenesis of sepsis [20]. Therefore, the role of *TUG1* in macrophages was further investigated. Overexpression of *TUG1* increased the ratio of macrophages CD68^+^ and F4/80^+^/total macrophages, elevated ratio of M1 macrophage iNOS and TNF-α/total macrophages, while reduced ratio of M2 macrophage CD206/total macrophages in the septic mice (Fig. 2A; Supplementary Fig. 1). Subsequently, macrophages were induced with LPS in vitro. The results presented that 24 h of LPS stimulation led to increased mRNA expression of the M1 markers iNOS and TNF-α (Fig. 2B), as well as elevated M2 markers IL-10 and Arg-1 (Fig. 2C), level of TNF-α in the macrophages (Fig. 2D), and expression of iNOS in the medium (Fig. 2E). In contrast, treatment with Ad-*TUG1* reduced the expression of M1 markers iNOS and TNF-α, but increased the expression of M2 markers IL-10 and Arg-1 in LPS-exposed macrophages and the corresponding medium (Fig. 2D, E).

### EPCs derived EVs carrying *TUG1* into macrophages promotes macrophage M2 polarization

**Fig. 3:**
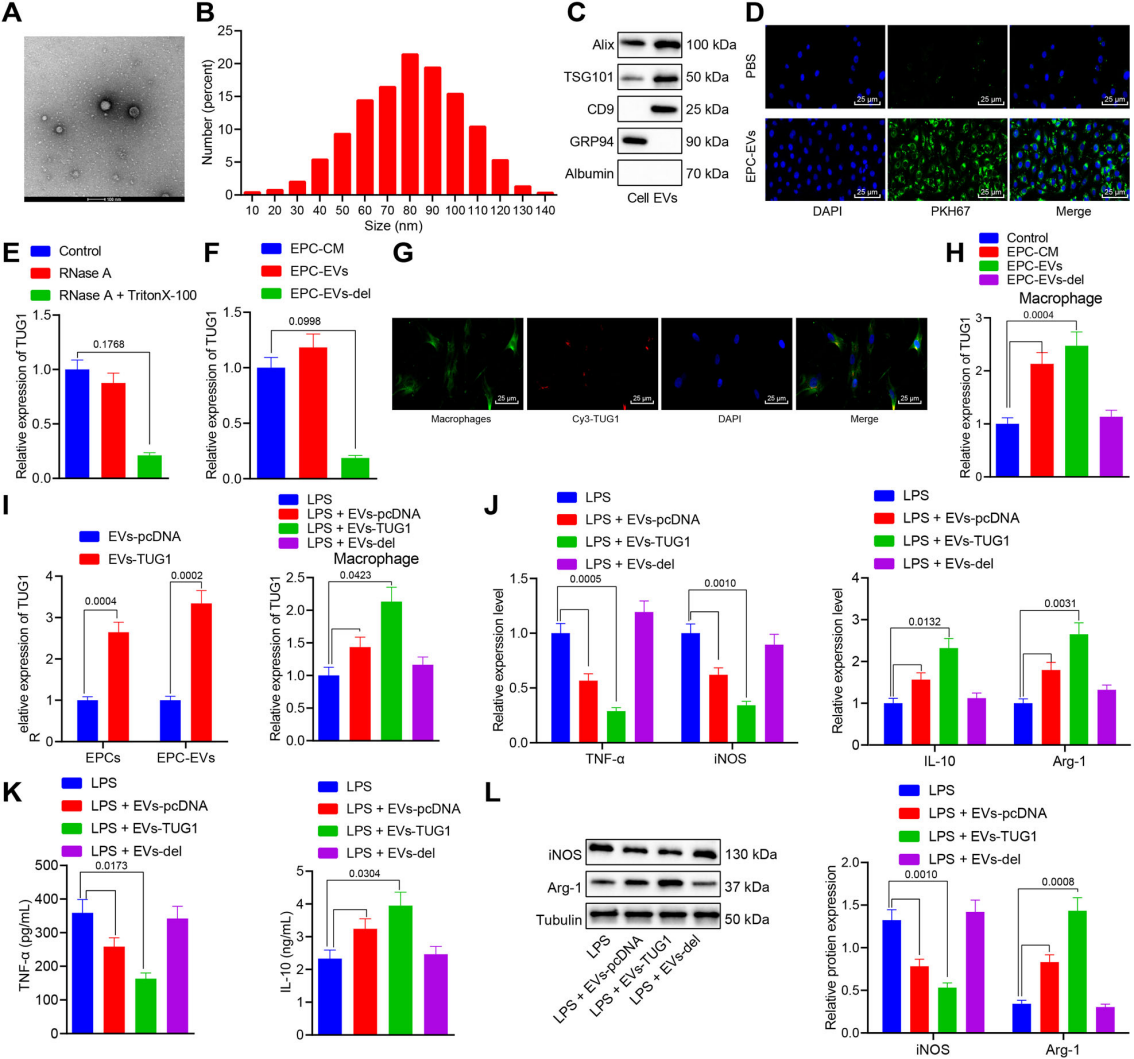
*TUG1* in EPCs can be transmitted to macrophages through EVs and then promotes macrophage M2 polarization. **A** Particle diameter of EPC-derived EVs (bar = 100 nm). **B** The diameter distribution and concentration of EVs detected by NTA. **C** Western blot analysis for determination of protein expression of ALIX, TSG101, CD9, GRP94, and albumin. **D** Uptake of EPC-derived EVs labeled with fluorescent PKH67 by macrophages observed by immunofluorescence microscopy. The PKH67-labeled EVs were red-colored and the DAPI-stained nuclei were blue (scale bar = 25 μm). **E** The level of *TUG1* measured by RT-qPCR after RNase treatment alone. **F** The expression of *TUG1* in EPCs derived EVs and EPC medium determined by RT-qPCR. **G** Uptake of EPC-derived EVs labeled with Cy3-TUG1 by macrophages observed by immunofluorescence microscopy. The Cy3-TUG1 labeled EVs were red, the DAPI-stained nuclei were blue, and phalloidin labeled macrophages were green (scale bar = 25 μm). **H** The expression of *TUG1* in macrophages cocultured with EPCs derived EVs determined by RT-qPCR. **I** The expression of *TUG1* in EPCs and EPCs derived EVs after pcDNA TUGq-transfected EPCs cocultured with macrophages assessed by RT-qPCR. **J** RT-qPCR for measurement of mRNA expression of iNOS, TNF-α, IL-10, and Arg-1 in macrophages cocultured with EPCs derived EVs. **K** The contents of IL-10 and TNF-α protein in the cell culture medium evaluated by ELISA. **L** The protein expression of iNOS and Arg-1 in macrophages cocultured with EPCs derived EVs measured by western blot analysis. Measurement data were expressed as mean ± standard deviation. Data between two groups were compared using unpaired *t*-test. One-way ANOVA was adopted for comparison between multiple groups followed by Tukey’s post hoc test. Cell experiments were conducted three times independently. *p < 0.05 vs. cells, control, EPC-CM, EVs-pcDNA, or LPS group.

It has been reported that EPCs derived EVs can prevent microvascular dysfunction and thus potentially prevent sepsis [7, 21], yet the underlying mechanism remains undefined. EPCs were isolated and purified from the mouse umbilical cord blood samples (Supplementary Fig. 2), and EPCs derived EVs were isolated from the cell supernatant. We observed that the isolated EPCs derived EVs were double-layer membrane vesicles with a concentration of about 1.6 × 10^11^ particles/mL; the diameter of 94% of the isolated particles ranged from 30 to 120 nm (Fig. 3A, B). Western blot analysis results confirmed the presence of ALIX, TSG101, and CD9 in the EPCs derived EVs, and presented with negative staining for non-EVs markers such as GRP94 and vital serum contaminants such as albumin in the supernatant (Fig. 3C).

After 12 h of culture of PKH67-labeled EPCs derived EVs with the macrophages, the macrophages were labeled with PKH67 and photographed (Fig. 3D), showing that EPCs derived EVs entered macrophages and distributed around the nucleus. The website (http://www.EVsrbase.org) showed that *TUG1* existed in the circulating EVs, as well as in a variety of living cells. After RNase treatment alone, the level of *TUG1* in the medium remained unchanged, whereas the simultaneous treatment with RNase and Triton X-100 led to decreased level of *TUG1*, indicating that *TUG1* was enveloped by the membrane and not directly released (Fig. 3E). In addition, the expression of *TUG1* in the EPCs derived EVs was equivalent to that in the EPC medium, but after removal of EVs from the medium, *TUG1* was almost undetectable (Fig. 3F), suggesting that EVs were the primary carriers of *TUG1*. EPCs were transfected with red fluorescent Cy3-labeled TUG1. After 48 h, the cell supernatant was collected and ultracentrifuged to obtain EVs, which were then cocultured with macrophages for 12 h. The images displayed that Cy3-labeled TUG1 was localized in the cytoplasm of macrophages (Fig. 3G).

RT-qPCR suggested that, compared with the macrophages treated with medium alone (EPC-CM group), the expression of *TUG1* was elevated in macrophages after coculture with EPCs derived EVs (EPC EVs group). However, no significant difference was evident in the expression of *TUG1* in macrophages cultured in EVs depleted EPC medium (EPC–EVs del group) (Fig. 3H). The preceding results demonstrated that *TUG1* in EPCs could be transmitted to the macrophages by EVs. Besides, the results revealed that *TUG1* expression was notably increased in the transfected EPCs and their derived EVs (Fig. 3I). Macrophages were cocultured with EPCs and stimulated with LPS. The results showed that treatment with EPCs derived EVs increased *TUG1* expression in LPS-exposed macrophages and the expression of IL-10 and Arg-1, but diminished iNOS and TNF-α expression. The increase in the expression of M2 markers and reduction in expression of M1 markers were of greater magnitude in the LPS-exposed macrophages cocultured with the EPCs overexpressing TUG1. However, the coculture of macrophages with EPC–EVs del induced no significant change in those markers (Fig. 3I–L).

### *TUG1* upregulates the expression of SIRT1 by binding to miR-9-5p

**Fig. 4:**
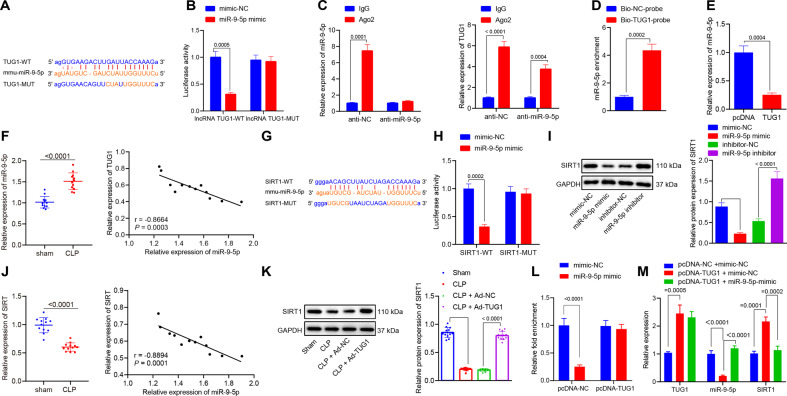
*TUG1* upregulates the expression of SIRT1 by functioning as a ceRNA of miR-9-5p. **A** Complementary binding sites and mutation sites between *TUG1* and miR-9-5p predicted on Starbase website (http://starbase.sysu.edu.cn/). **B** The luciferase activity of *TUG1*-WT and MUT analyzed by dual-luciferase reporter gene assay. **C** Enrichment of *TUG1* and miR-9-5p by anti-Ago2 or anti-IgG analyzed by RIP assay. **D** *TUG1* pull-down pellet containing a large amount of miR-9-5p examined by RNA pull-down assay. **E** The level of miR-9-5p in macrophages transfected with pcDNA-*TUG1* or pcDNA-NC determined by RT-qPCR. **F** The relative expression of miR-9-5p in sham- or CLP-operated mice evaluated by RT-qPCR (left), and Pearson’s correlation analysis of the levels of *TUG1* and miR-9-5p in mouse lung tissues (right, *n* = 12). **G** SIRT1 as a downstream target of miR-9-5p predicted on Starbase, showing the putative SIRT1 binding site of miR-9-5p (SIRT1-WT) and the designed mutant sequence (SIRT1-MUT). **H** The luciferase activity examined by the dual-luciferase reporter gene 48 h after cotransfection with miR-9-5p mimics and SIRT1-WT or SIRT1-MUT. **I** The protein expression of SIRT1 in in macrophages transfected with miR-9-5p mimic, miR-9-5p inhibitor, or their NCs determined by western blot analysis. **J** The relative expression of SIRT1 in sham- or CLP-operated mice assessed by RT-qPCR (left), and the Pearson’s correlation analysis of the levels of SIRT1 and miR-9-5p in the mouse lung tissues (right, *n* = 12). **K** Western blot analysis for determination of protein expression of SIRT1 in mouse lung tissues. **L** The binding of miR-9-5p and SIRT1 after pcDNA-*TUG1* treatment identified by dual-luciferase reporter gene assay. **M** *TUG1*, miR-9-5p, and SIRT1 expression after treatment with pcDNA-*TUG1* and miR-9-5p mimic were determined by RT-qPCR. Measurement data were expressed as mean ± standard deviation. Data between two groups were compared using unpaired *t*-test. One-way ANOVA was adopted for comparison between multiple groups followed by Tukey’s post hoc test. Cell experiments were conducted three times independently. *p < 0.05 vs. mimic NC, IgG, Bio-NC-probe, pcDNA, or sham group; ^#^*p* < 0.05 vs. anti-NC, inhibitor-NC, CLP + Ad-NC, or pcDNA-*TUG1* + mimic NC group.

To identify the downstream regulatory mechanism of *TUG1* in sepsis, Starbase website was adopted, which consistently predicted the complementary binding sites between *TUG1* and miR-9-5p (Fig. 4A). The binding sites were mutated, and dual-luciferase reporter gene assay, RIP, and RNA pull-down experiments were utilized to identify *TUG1* as a ceRNA of miR-9-5p. The dual-luciferase reporter gene assay showed that overexpression of miR-9-5p inhibited the luciferase activity of *TUG1*-WT but did not affect that of *TUG1*-MUT (Fig. 4B). The results of RIP displayed that the *TUG1* expression was abundantly present in the Ago2 immunoprecipitated complex in the control group, while expression in the Ago2 complex purified from cells treated with miR-9-5p inhibitor was reduced (Fig. 4C), indicating that *TUG1* may indeed exist in the miR-9-5p RISC complex. Results of RNA pull-down assay revealed enrichment of miR-9-5p in the *TUG1* pull-down pellet (Fig. 4D), which suggested that miR-9-5p could recognize the TUG1 sequence. miR-9-5p expression in the macrophages transfected with pcDNA-*TUG1* was reduced (Fig. 4E). The tissues from septic mice revealed higher miR-9-5p levels than those from the sham-operated mice and the expression of *TUG1* in the lung tissues of septic mice was negatively correlated with miR-9-5p (Fig. 4F). Altogether, *TUG1* could competitively bind to miR-9-5p to regulate its expression. Subsequently, we explored whether *TUG1* could regulate the target gene of miR-9-5p by functioning as ceRNA. Through the Starbase website, the binding sites were predicted between miR-9-5p and SIRT1, as reported in previous work [22, 23]. Hence, we speculated that miR-9-5p interacted with SIRT1 to affect sepsis. We designed WT-SIRT1 and MUT-SIRT1 sequences and measured the luciferase activity to evaluate the binding ability (Fig. 4G). The results of dual-luciferase reporter gene assay showed that the luciferase activity of WT-SIRT1 but not MUT-SIRT1 was lowered by miR-9-5p mimic (Fig. 4H). In addition, the SIRT1 level was reduced in macrophages transfected with the miR-9-5p mimic but increased in macrophages transfected with the miR-9-5p inhibitor (Fig. 4I). Moreover, SIRT1 levels in the lung tissues of septic mice were reduced, and the miR-9-5p level was negatively correlated with the SIRT1 expression (Fig. 4J). To exclude the possibility of effects caused by other miRs, we adopted RT-qPCR and western blot analysis to detect the expression of miR-204 in LPS-induced macrophages, which revealed that overexpression of miR-204 increased the expression of miR-204 but had no significant effect on the expression of SIRT1 in macrophages (Supplementary Fig. 3A, B). The above results indicated that the expression of SIRT1 was specifically regulated by miR-9-5p among the downstream miRNAs regulated by *TUG1*. Western blot analysis results demonstrated that protein expression of SIRT1 in mice was reduced after CLP treatment, but this effect could be reversed by injection of Ad-*TUG1* (Fig. 4K). After pcDNA-*TUG1* was transfected into the macrophages, dual-luciferase reporter gene assay verified enhanced binding of miR-9-5p to SIRT1 (Fig. 4L). RT-qPCR results further revealed that overexpression of *TUG1* decreased miR-9-5p expression and increased SIRT1 expression, while the miR-9-5p mimic treatment reduced the SIRT1 expression in the presence of *TUG1* (Fig. 4M). Subsequently, the protein levels of SIRT2, SIRT3, and SIRT6 were determined using western blot analysis, which demonstrated that elevation of TUG1 brought about no difference in SIRT2, SIRT3, and SIRT6 levels (Supplementary Fig. 4A, B).

### EPCs derived EVs containing *TUG1* upregulates SIRT1 expression by binding to miR-9-5p to promote macrophages M2 polarization

**Fig. 5:**
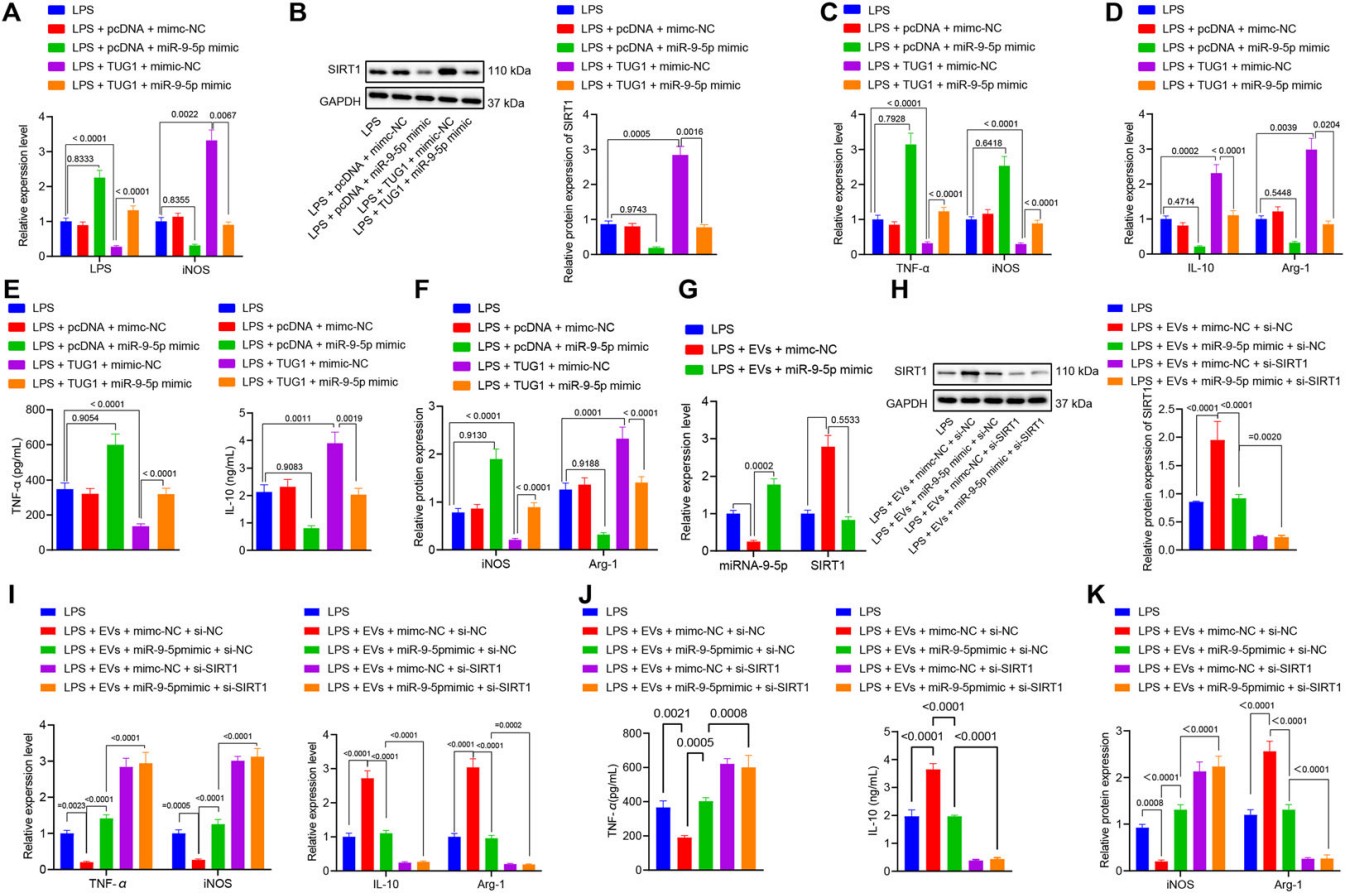
EPC-derived EVs carrying *TUG1* stimulates macrophages M2 polarization by upregulating SIRT1 expression through inhibition of miR-9-5p. **A** The expression of miR-9-5p and SIRT1 in macrophages transfected with pcDNA-*TUG1* or miR-9-5p mimic or their NCs determined by RT-qPCR. **B** Western blot analysis for determination of protein expression of SIRT1 in macrophages transfected with pcDNA-*TUG1* or miR-9-5p mimic or their NCs. **C** RT-qPCR for measurement of mRNA expression of iNOS and TNF-α in macrophages transfected with pcDNA-*TUG1* or miR-9-5p mimic or their NCs **D** RT-qPCR for measurement of the mRNA expression of IL-10 and Arg-1 in macrophages transfected with pcDNA-*TUG1* or miR-9-5p mimic or their NCs. **E** IL-10 and TNF-α contents in the medium detected by ELISA. **F** The protein expression of iNOS and Arg-1 in macrophages transfected with pcDNA-*TUG1* or miR-9-5p mimic or their NCs measured by western blot analysis. **G** RT-qPCR for determination of mRNA expressions of miR-9-5p and SIRT1 in macrophages cocultured with EPCs derived EVs measured by. **H** The protein expression of SIRT1 in macrophages cocultured with EPCs derived EVs examined by western blot analysis. **I** Determination of the mRNA expression of iNOS, TNF-α, IL-10, and Arg-1 in macrophages cocultured with EPCs derived EVs by RT-qPCR. **J** The levels of IL-10 and iNOS in the medium assessed by ELISA. **K** Measurement of the protein expression of iNOS and Arg-1 in macrophages cocultured with EPCs derived EVs by western blot analysis. Measurement data were expressed as mean ± standard deviation. One-way ANOVA was adopted for comparison between multiple groups followed by Tukey’s post hoc test. Cell experiments were conducted three times independently. *p < 0.05 vs. LPS group; ^#^*p* < 0.05 vs. LPS + EVs + mimc-NC or inhibitor-NC or LPS + *TUG1* + mimic NC group.

Macrophages were transfected with pcDNA-*TUG1* or miR-9-5p mimic or their NCs, and then treated with LPS. After LPS stimulation for 24 h, the expression of miR-9-5p and SIRT1 in the macrophages was determined by RT-qPCR and western blot analysis. The results demonstrated that miR-9-5p mimic increased miR-9-5p expression and decreased SIRT1 expression, while upregulation of *TUG1* reduced miR-9-5p expression and elevated the SIRT1 expression. *TUG1* could restore the expression of SIRT1 in the presence of miR-9-5p (Fig. 5A, B). The mRNA expression of iNOS and TNF-α in the macrophages and the protein expression of TNF-α in the medium and that of iNOS in macrophages were increased, while the expression of IL-10 and Arg-1 was reduced by the miR-9-5p mimic, the effect of which was counteracted in response to *TUG1* overexpression (Fig. 5C–F). These results showed that *TUG1* impaired miR-9-5p-dependent inhibition of SIRT1 to promote the polarization of macrophages to the anti-inflammatory M2 state. Macrophages transfected with miR-9-5p mimic or its NC or si-SIRT1 were cocultured with EPC-derived EVs. The results revealed that treatment with miR-9-5p mimic elevated miR-9-5p expression, and reduced levels of SIRT1 in the macrophages (Fig. 5G, H). The mimic treatment also resulted in increased mRNA expression of iNOS and TNF-α (Fig. 5I), upregulated protein expression of TNF-α in the medium and iNOS in macrophages (Fig. 5J, K), and diminished expression of IL-10 and Arg-1 (Fig. 5I–K). Compared with the EVs + mimic NC + si-SIRT1 group, the EVs + miR-9-5p mimic + si-SIRT1 group showed unchanged expression of SIRT1, TNF-α, iNOS, IL-10, and Arg-1; compared with the LPS + EVs + miR-9-5p mimic + si-NC group, the LPS + EVs + miR-9-5p mimic + si-SIRT1 group showed increased TNF-αand iNOS expression but decreased IL-10 and Arg-1 (Fig. 5G–K). This showed that when the SIRT1 gene was silenced, miR-9-5p could not affect the polarization of macrophages due to the lack of its target genes. The results indicated that these effects were dependent on the presence of SIRT1.

### EPC-derived EVs harboring *TUG1* accelerates lung macrophage M2 polarization

**Fig. 6:**
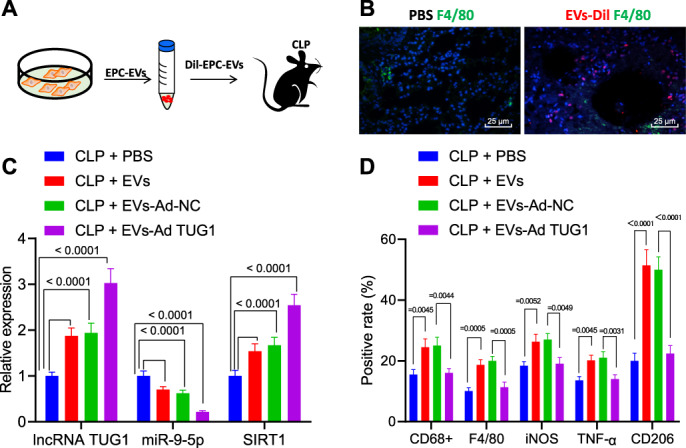
EPC EVs encapsuled *TUG1* promotes macrophage M2 polarization in the murine model. **A** Flow chart of injection of EPC-derived EVs or PBS in CLP-stimulated septic mice. **B** Localization of Dil staining (red) prelabeled EVs and F4/80-labeled macrophages (green) in lung tissues (scale bar = 25 μm). **C** The expression of *TUG1*, miR-9-5p, and SIRT1 in the lung tissues of CLP-induced septic mice injected with EPC-derived EVs determined by RT-qPCR (*n* = 12). **D** The number of macrophages CD68^+^ and F4/80^+^ evaluated by flow cytometry and percentages of M1 markers iNOS and TNF-α as well as M2 marker CD206 in a single-cell suspension of whole lung tissues from the mice (*n* = 12). Measurement data were expressed as mean ± standard deviation. One-way ANOVA was adopted to analyze comparison between multiple groups with Tukey’s post hoc test. One-way ANOVA was adopted for comparison between multiple groups followed by Tukey’s post hoc test. *p < 0.05 vs. CLP + PBS group; ^#^*p* < 0.05 vs. CLP + EVs + Ad-NC group.

The impact of *TUG1*/miR-9-5p/SIRT1 on macrophages was further verified in the CLP-induced septic mice. Dil (red) was applied to prelabel the EVs (Fig. 6A). EPC-derived EVs or PBS were injected intravenously into mice 4 h after CLP surgery. Six hours after injection of Dil-labeled EPC-derived EVs, accumulation of F4/80 (green)-labeled macrophages was observed in the lung epithelial cells of CLP-operated mice (Fig. 6B). RT-qPCR revealed that *TUG1* and SIRT1 levels were increased but miR-9-5p expression was decreased in the lung tissues of the CLP-induced septic mice injected with EPCs derived EVs, and the impacts were increased by EVs from EPCs treated with Ad-*TUG1* (Fig. 6C). In addition, EPC-derived EVs effectively led to a reduced number of macrophages (CD68^+^ and F4/80^+^), reduced iNOS, and TNF-α expression, and increased CD206 signal in the lung tissues of CLP-induced septic mice; more significant changes were detected when the mice injected with EVs from EPCs overexpressing *TUG1* (Fig. 6D).

### Protective effect of EPCs–EVs encapsuled *TUG1* against sepsis-induced organ damage and in murine model

**Fig. 7:**
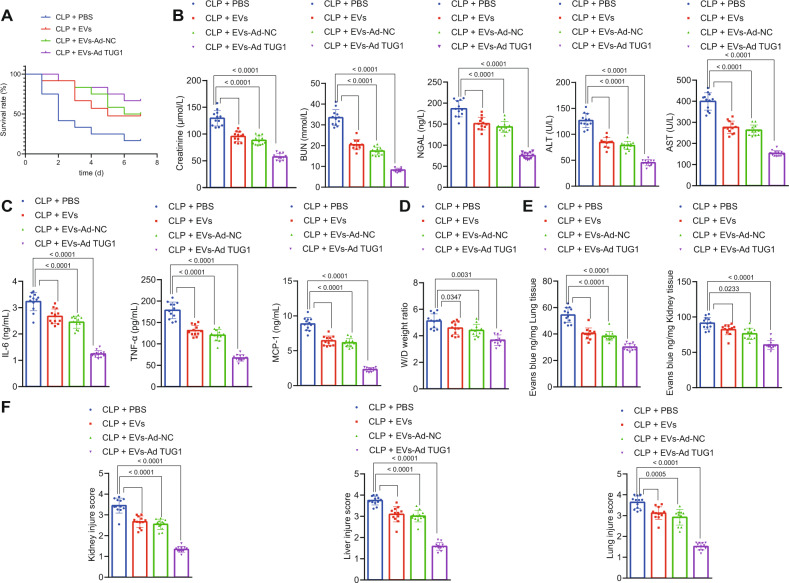
EPC-derived EVs carrying *TUG1* alleviate multiple organ damage induced by sepsis. **A** The survival rate of mice within 7 days after CLP analyzed by Kaplan–Meier analysis. **B** Measurement of creatinine, BUN, NGAL, AST, and ALT levels in mouse serum. **C** Serum levels of IL-6, TNF-α, and MCP-1 assessed by ELISA 24 h after surgery. **D** Pulmonary edema in mice assessed by the wet/dry ratio. **E** The measurement of blood vessel leakage in lung and liver of mice by injecting Evans blue dye. **F** The morphological changes of lung, liver, and kidney tissues observed by HE staining 24 h after CLP. Measurement data were expressed as mean ± standard deviation. One-way ANOVA was adopted for comparison between multiple groups followed by Tukey’s post hoc test. Kaplan–Meier analyses were performed to calculate the survival rate. Log-rank test was conducted for univariate survival analysis. *p < 0.05 vs. sham-operated mice; ^#^*p* < 0.05 vs. CLP-operated mice treated with Ad-NC (*n* = 12).

The effect of EPC-derived EVs carrying *TUG1* on multiple organ damage in sepsis was further investigated in vivo. Injection of EPC-derived EVs notably elevated the 7-day survival rate of CLP-induced septic mice, while injection of EPC-derived EVs carrying upregulated *TUG1* contributed to a higher survival rate (Fig. 7A). ELISA detection of cytokines and chemokines in the mouse plasma suggested that treatment with EPC-derived EVs reduced the elevation of creatinine, BUN, NGAL, AST, and ALT levels (Fig. 7B) as well as IL-6, TNF-α, and MCP-1 contents (Fig. 7C) and diminished pulmonary edema (Fig. 7D) as well as pulmonary and renal vascular leakage (Fig. 7E) in the CLP-induced septic mice. Hence, the kidney, liver, and lung dysfunction in the CLP-induced septic mice was alleviated by EPC-derived EVs. Furthermore, the injection of EPC-derived EVs with a high expression of *TUG1* exerted protective effects against kidney, liver, and lung dysfunction (Fig. 7B–E). Histological staining results displayed reduced visible tubules in the outer kidney medulla, inflammatory cell infiltration, and loss of brush border epithelium in the kidney, and suppressed vacuolation in the liver tissues and in the outer medullary strips of kidney tissues in the septic mice injected with EPC-derived EVs (Fig. 7F). Less damage was observed in the kidney, liver, and lung tissues of septic mice injected with EPC-derived EVs carrying a high *TUG1* expression.

## Discussion

**Fig. 8:**
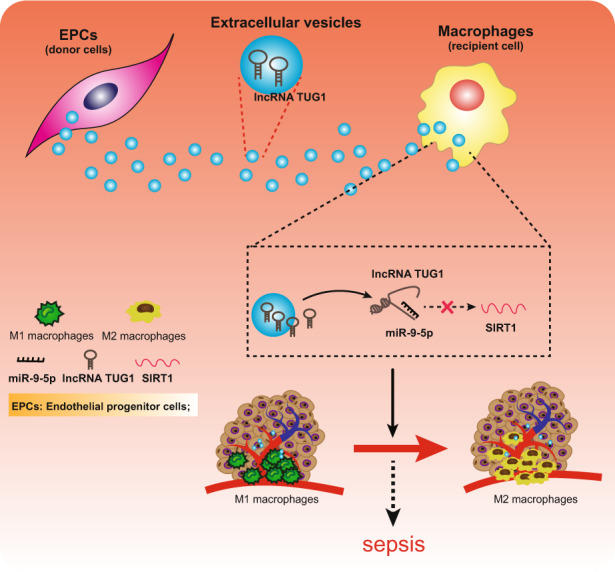
Schematic representation and function of *TUG1*/miR-9-5p/SIRT1 axis in the regulation of macrophage polarization in sepsis. EVs encapsuled *TUG1* from EPCs stimulated the M2 macrophage polarization and inhibited inflammation by upregulating SIRT1 expression through inhibition of miR-9-5p to prevent the development of sepsis.

The current study suggested that *TUG1* was downregulated in sepsis and that its upregulation contributes to improved protection against the sepsis-caused inflammatory damage by impairing miR-9-5p-targeted inhibition of SIRT1 (Fig. 8).

We found that *TUG1* exerted an anti-inflammatory function in sepsis. Consistently, *TUG1* gain of function can protect against LPS-caused inflammatory injury [24]. Also, *TUG1* suppresses the secretion of inflammatory proteins such as IL-1β, IL-6, and IL-8 in colon HT-29 cells exposed to TNF-α treatment, demonstrating its anti-inflammatory role [25]. Besides, our study demonstrated the contributory role of *TUG1* to the polarization of M1 to M2 macrophages, which has been scarcely reported. Essentially, our in vivo murine model validated the protective role of *TUG1* against sepsis-evoked liver damage. A previous study indicated the ability of TUG1 to impair miR-34b-5p-mediated down-regulation of GAB1, thereby exercising a preventive effect against sepsis-induced acute kidney injury [12]. In light of the preceding evidence, we set out to investigate the *TUG1*-mediated anti-inflammatory mechanism in the sepsis models.

Our data also highlighted that *TUG1* could bind to miR-9-5p to radically upregulate the expression of the miR-9-5p target gene SIRT1. A recent miRNA–mRNA integrated analysis revealed miR-9-5p as a miRNA enriched in M1 macrophages expressing diverse genes mediating fundamental immune responses [26]. Besides, the neurotoxic and proinflammatory roles of miR-9-5p have been highlighted in Parkinson’s disease by inhibiting SIRT1 [22]. However, their corresponding function in sepsis remains yet to be elucidated. SIRT1 is one of the well-characterized stress adapters and epigenetic enzymes from the sirtuin family, known for its protective action in cellular processes such as inflammation, vascular aging, and cardiac diseases [27]. SIRT1 potentially ameliorates inflammation induced by monosodium urate crystal deposition by modulating macrophage polarization [28]. SIRT1 can hinder the acetylation of NICD to restrain activation of Notch signaling to subsequently alleviate sepsis [29]. The specific molecules related to SIRT1-mediated macrophage polarization in sepsis warrants further investigation.

Finally, our findings substantiated that *TUG1* was enriched in EPCs derived EVs and could be transferred to macrophages, hence stimulating macrophage polarization. EVs secreted from different cells have emerged as vital carriers of specific diagnostic and therapeutic molecules for diverse biological functions. For instance, mesenchymal stromal cell-derived EVs can effectively alleviate sepsis due to their favorable immunogenicity and safety profile [30]. In contrast, plasma EVs can induce the inflammatory response through miR- and TLR7-dependent mechanisms [31]. Consistent with our findings, EPCs derived EVs ameliorated sepsis outcomes, which were potentially induced via delivery of miR-126 [8]. Our study additionally substantiated the protective role of EPC EVs encapsuled *TUG1* against sepsis in the murine model.

To conclude, the current study gives evidence for the contribution of *TUG1* to macrophage polarization and its anti-inflammatory potency by blocking miR-9-5p-induced silencing of SIRT1 in sepsis. Hence, EVs encapsuled *TUG1* presents a potential strategy for the clinical treatment of sepsis. However, due to the challenges in the isolation and purification of EVs, more investigations are necessary for the development of EV-based therapy. Thus, the translation of this finding into clinical application requires further validation.

## Supplementary information

Supplementary figure legends

Supplementary Figure 1

Supplementary Figure 2

Supplementary Figure 3

Supplementary Figure 4

Supplementary Tables

aj-checklist

cddis-author-contribution-form
