# Supplementary material for: Functional delivery of lncRNA TUG1 by endothelial progenitor cells derived extracellular vesicles confers anti-inflammatory macrophage polarization in sepsis via impairing miR-9-5p-targeted SIRT1 inhibition
Source: Cell Death Dis. 2021 Nov 6;12(11):1056. doi: 10.1038/s41419-021-04117-5 (PMC8572288; doi:10.1038/s41419-021-04117-5)
Supplement: Supplementary file 1 — Supplementary figure legends [file 41419_2021_4117_MOESM1_ESM.docx]

**Supplementary figure legends**

**Supplementary Fig. 1.** The graphs of number of CD68^+^ and F4/80^+^ macrophages and percentages of M1 markers iNOS and TNF-α as well as M2 marker CD206 in a single-cell suspensions of whole lung tissue from the mice after *TUG1* overexpression (n = 12).

**Supplementary Fig. 2.** Identification of EPCs. A, Observation of the morphological changes of EPCs under an inverted microscope. After 48 h of inoculation, some cells adhered to the cells and the cell body increased in size, with enhanced transillumination of the adherent cells. After 10 days after inoculation, most of cells were short fusiform, the cell size increased, the adherent cells were close to fusion, and the fusion cells showed a paving-stone-like growth pattern (scale bar = 50 μm). B, Angiogenesis in EPCs (scale bar = 50 μm). C, Dual-fluorescence staining in the cells with stable uptake of ac-LDL observed under inverted fluorescence microscope, and the cells bound to lectin UEA-1 were differentiated into EPCs. The EPCs stably internalized ac-LDL were observed with red signal under the inverted fluorescence microscope. EPCs bound to lectin UEA-1 displayed green. EPCs counter-stained with Hoechst turned blue (scale bar = 25 μm). D, EPC markers detected by immunofluorescence. EPCs showed stable expression of specific antigens CD133 (red), CD34 (green), vegfr-2 (red), v WF (green) and EPCs counter-stained with hoechst3342 displayed blue (scale bar = 50 μm).

**Supplementary Fig. 3.** Overexpression of miR-204 exerts no effect on SIRT1 expression in macrophages. A, The expression of miR-204 and SIRT1 in macrophages determined with RT-qPCR; B, The expression of miR-204 and SIRT1 in macrophages determined with Western blot analysis. Measurement data were expressed as mean ± standard deviation. Data between two groups were compared using unpaired *t*-test. ***p* < 0.01.

**Supplementary Fig. 4.** Overexpression of *TUG1* exerts no effect on SIRT2, SIRT3 and SIRT6 expression in macrophages. The protein expression of SIRT2, SIRT3 and SIRT6 in macrophages determined with Western blot analysis. Measurement data were expressed as mean ± standard deviation. Data between two groups were compared using unpaired *t*-test. ***p* < 0.01.
