# Supplementary material for: Functional delivery of lncRNA TUG1 by endothelial progenitor cells derived extracellular vesicles confers anti-inflammatory macrophage polarization in sepsis via impairing miR-9-5p-targeted SIRT1 inhibition
Source: Cell Death Dis. 2021 Nov 6;12(11):1056. doi: 10.1038/s41419-021-04117-5 (PMC8572288; doi:10.1038/s41419-021-04117-5)
Supplement: Supplementary file 6 — Supplementary Tables [file 41419_2021_4117_MOESM6_ESM.docx]

**Supplementary Table 1** Primer sequences for RT-qPCR

| Target | Primer sequence |
| --- | --- |
| *iNOS* | F: 5’-GTTCTCAGCCCAACAATACAAGA-3’ |
|  | R: 5’-GTGGACGGGTCGATGTCAC-3’ |
| *Arg-1* | F: 5’-CTCCAAGCCAAAGTCCTTAGAG-3’ |
|  | R: 5’-AGGAGCTGTCATTAGGGACAT-3’ |
| *GAPDH* | F: 5’-AGGTCGGTGTGAACGGATTTG-3’ |
|  | R: 5’-GGGGTCGTTGATGGCAACA-3’ |
| miR-9-5p | F: 5’-CGCGCTCTTTGGTTATCTAGCTGTA-3’ |
|  | R: 5’-GTGCAGGGTCCGAGGTATTC-3’ |
| LncRNA *TUG1* | F: 5’-CATCTCACAAGGCTTCAACCA-3’ |
|  | R: 5’-ACCTCAACTCCCACTTCACTA-3’ |
| *U6* | F: 5’-CTCGCTTCGGCAGCACA-3’ |
|  | R: 5’-AACGCTTCACGAATTTGCGT-3’ |
| *Il-10* | F: 5’-CTTACTGACTGGCATGAGGATCA-3’ |
|  | R: 5’-GCAGCTCTAGGAGCATGTGG-3’ |
| *TNF-α* | F: 5’-TATGGCCCAGACCCTCACA-3’ |
|  | R: 5’-GGAGTAGACAAGGTACAACCCATC-3’ |
| *SIRT1* | F: 5’-CAGACCCTCAAGCCATGTTTGATA-3’ |
|  | R: 5’-TTGGATTCCTGCAACCTGCTC-3’ |

Note: RT-qPCR, reverse transcription quantitative polymerase chain reaction; iNOS, inducible nitric oxide synthase; Arg-1, arginase 1; GAPDH, glyceraldehyde 3-phosphate dehydrogenase. miR-9-5p, microRNA-9-5p. lncRNA *TUG1*, long non-coding RNA taurine upregulated gene 1; IL-10, Interleukin 10; TNF-α, tumor-necrosis factor-α; SIRT1, sirtuin 1; F, forward; R, reverse.

**Supplementary Table 2** Used antibodies for Western blot analysis

| Antibodies | ID | Company |
| --- | --- | --- |
| Tubulin | ab6046 | Abcam |
| GAPDH | ab8245 | Abcam |
| SIRT1 | ab189494 | Abcam |
| SIRT2 | ab51023 | Abcam |
| SIRT3 | ab217319 | Abcam |
| SIRT6 | ab17435 | Abcam |
| iNOS | ab178945 | Abcam |
| Arg-1 | ab124917 | Abcam |
