# Supplementary material for: Functional delivery of lncRNA TUG1 by endothelial progenitor cells derived extracellular vesicles confers anti-inflammatory macrophage polarization in sepsis via impairing miR-9-5p-targeted SIRT1 inhibition
Source: Cell Death Dis. 2021 Nov 6;12(11):1056. doi: 10.1038/s41419-021-04117-5 (PMC8572288; doi:10.1038/s41419-021-04117-5)
Supplement: Supplementary file 8 — cddis-author-contribution-form [file 41419_2021_4117_MOESM8_ESM.pdf]

DECLARATION OF CONTRIBUTIONS TO ARTICLE

ADMC

Manuscript Number: CDDIS-20-4793R

Journal Name: *Cell Death & Disease* (the 'Journal')

Proposed Title of the Contribution:

Functional delivery of lncRNA TUG1 by endothelial progenitor cells-derived extracellular vesicles confers anti-inflammatory macrophage polarization in sepsis via impairing miR-9-5p-targeted SIRT1 inhibition (the 'Contribution)

Author(s):

Wentao Ma 1#, Weihong Zhang 2#, Bing Cui 3, Jing Gao 1, Qiuhong Liu 1, Lihua Xing 1*, Hanbing Ning 4*, Mengying Yao 1* (the 'Authors')

For all *CDDis* articles, each person named as an author in the published version must be able to show he or she has contributed substantially to the article.

Authorship credit should be based on 1) substantial contributions to conception and design, acquisition of data, or analysis and interpretation of data; 2) drafting the article or revising it critically for important intellectual content; and 3) final approval of the version to be published. Authors should meet conditions 1, 2 and 3.

Any person who cannot be shown to have made a substantial contribution to the article cannot be listed as an author in the final version. The name of any person who is deemed to have made a minor contribution can, however, appear in the Acknowledgments section of the article.

Please complete the table below to indicate the contributions of all named authors to the manuscript.

Author Full Name:	Specification of Contribution to the Manuscript:
Wentao Ma	contributed to the acquisition of data;contributed to drafting the article;contributed to revising the article critically for important intellectual content;approved the final version to be submitted
Weihong Zhang	contributed to the conception and design of the study;contributed to the analysis and interpretation of data;approved the final version to be submitted
Bing Cui	contributed to the conception and design of the study;contributed to the acquisition of data;approved the final version to be submitted
Jing Gao	contributed to revising the article critically for important intellectual content;approved the final version to be submitted
Qiuhong Liu	contributed to revising the article critically for important intellectual content;approved the final version to be submitted
Lihua Xing	contributed to drafting the article;approved the final version to be submitted
Hanbing Ning	contributed to revising the article critically for important intellectual content;approved the final version to be submitted
Mengying Yao	contributed to the conception and design of the study;contributed to the acquisition of data;contributed to the analysis and interpretation of data;approved the final version to be submitted

Please complete the table below to indicate the contributions of all named authors to the figures.

Figure 1:
Wentao Ma, Weihong Zhang

Figure 2:
Wentao Ma, Weihong Zhang

Figure 3:
Bing Cui, Jing Gao

Figure 4:
Lihua Xing, Hanbing Ning

Figure 5:
Lihua Xing, Mengying Yao

Figure 6:
Qiuhong Liu

Signed for and on behalf of the Author(s):	Print Name:	Date:
Lihua Xing	Lihua Xing	2021.4.26
